# First-line antiretroviral therapy and dyslipidemia in people living with HIV-1 in Cameroon: a cross-sectional study

**DOI:** 10.1186/1742-6405-8-33

**Published:** 2011-09-26

**Authors:** Eric Walter Pefura Yone, Awa Foueudjeu Betyoumin, André Pascal Kengne, François Jérome Kaze Folefack, Jeanne Ngogang

**Affiliations:** 1 Chest Unit of Yaounde Jamot Hospital, Cameroon; 2 Faculty of Medicine and Biomedical Sciences, University of Yaounde I, Cameroon; 3 Biochemistry laboratory of Yaounde University Center, Cameroon; 4 South African Medical Research Council & department of Medicine, University of Cape Town, Francie van Zijl Drive, Parow Valley, 7505 Cape Town, South Africa; 5 Medicine Unit of Yaounde General Hospital, Cameroon

**Keywords:** antiretroviral therapy, dyslipidemia, HIV, Cameroon

## Abstract

**Background:**

Data on lipid profile derangements induced by antiretroviral treatment in Africa are scarce. The aim of this study was to determine the prevalence and characteristics of lipid profile derangements associated with first-line highly active antiretroviral therapy (ART) among Cameroonians living with human immunodeficiency virus (HIV) infection.

**Methods:**

This cross-sectional study was conducted between November 2009 and January 2010, and involved 138 HIV patients who had never received ART (ART-naive group) and 138 others treated for at least 12 months with first line triple ART regimens that included nevirapine or efavirenz (ART group). Lipid profile was determined after overnight fast and dyslipidemia diagnosed according to the US National Cholesterol Education Program III criteria. Data comparison used chi-square test, Student t-test and logistic regressions.

**Results:**

The prevalence of total cholesterol ≥ 200 mg/dl was 37.6% and 24.6% respectively in ART group and ART-naive groups (p = 0.019). The equivalents for LDL-cholesterol ≥ 130 mg/dl were 46.4% and 21% (p ≤ 0.001). Proportions of patients with total cholesterol/HDL-cholesterol ratio ≥ 5 was 35.5% in ART group and 18.6% in ART-naive group (p ≤ 0.001). The distribution of HDL-cholesterol and triglycerides was similar between the two groups. In multivariable analysis adjusted for age, sex, body mass index, CD4 count and co-infection with tuberculosis, being on ART was significantly and positively associated with raised total cholesterol, LDL-cholesterol and TC/HDL cholesterol. The adjusted odd ratios (95% confidence interval, p-value) ART-treated vs. ART-naïve was 1.82 (1.06-1.12, p = 0.02) for TC ≥ 200 mg/dl; 2.99 (1.74-5.15), p < 0.0001) for LDL-cholesterol ≥ 130 mg/dl and 1.73 (1.04-2.89, p = 0.03) for TC/HDL-cholesterol ≥ 5.

**Conclusions:**

First-line antiretroviral therapy that includes nonnucleoside reverse transcriptase inhibitors is associated with pro-atherogenic adverse lipid profile in people with HIV-1 infection compared to untreated HIV-infected subjects in Yaounde. Lipid profile and other cardiovascular risk factors should be monitored in patients on such therapy so that any untoward effects of treatments can be optimally managed.

## Introduction

The advent of highly active antiretroviral therapy (HAART) has modified the natural history of human immunodeficiency virus (HIV) infection through reduction in risks of death associated with the condition and improvement of the quality of life of people living with the infection [1,2]. However, antiretroviral drugs also have side effects of varying order of severity. Derangements of lipid metabolism associated with HAART have been largely characterised in the West and in several developing countries, particularly in patients on treatment regimens including protease inhibitors (PIs) and stavudine [3,4], but also for treatment regimens including nevirapine and efavirenz [5,6]. Antiretroviral therapy (ART) can induce raised levels of total cholesterol (TC), LDL-cholesterol (LDL-c) and triglycerides (TG), and variables effects on HDL-cholesterol (HDL-c) levels [4]. These ART-induced lipid derangements are potentially atherogenic and can increase cardiovascular risk [7,8]. First-line HAART regimens as defined by the World Health Organisation (WHO) and that are largely used in resources-constrained countries do not include PIs [9,10]. Evidences in support of lipid profile derangements associated with HAART in sub-Saharan Africa are scarce [11,12]. The aim of the present study was to determine the prevalence and determinants of derangements in lipid profile associated with the use of first-line ART regimens in Cameroonians with HIV infection.

## Participants and Methods

### Study setting and Participants

This was a cross-sectional study. Participants were recruited between November 2009 and January 2010 at the registered treatment centre of the Yaounde Jamot's Hospital. Two groups of participants were selected. One group included individuals with HIV-1 infection and who had been receiving ART for at least 12 months, based on WHO first-line regimens (ART group). First-line ART regimens applied to these participants were those that included lamivudine (3TC), stavudine (d4T) or zidovudine (AZT), and nevirapine (NVP) or efavirenz (EFV). The choice of regimens was unrelated to potential factors that could induce a dyslipidemia, given that lipid profile assessment is not part of routine pre-ART treatment evaluation in this setting [9]. Patients who had had their treatment regimens changed during follow-up were excluded. The second group was made up of individuals newly diagnosed with HIV-1 infection and who were not yet receiving ART (ART-naïve group). Participants had to be at least 18 years of age and to have a treatment adherence rate ≥ 95% (for the treated group). Level of adherence was assessed by verbal administration of a standard series of questions adapted from Adult AIDS clinical trials group (AACTG) adherence instruments. The 95% rate of adherence is referable to 4-day recall data [13]. Participants were also required not to be on lipid modifying therapies at their enrolment. All participants gave their inform consent and the study was approved by the Cameroon National Ethic Committee (ref N°150/CNE/SE/09).

### Methods

For each participant, data were collected on the sociodemographic background, past medical history including the use of medications that could modify the lipid profile and active or history of tuberculosis. ART-naïve participants were screened for HIV-1 and HIV-2 infection with the use of a rapid test (DETERMINE HIV 1-2, Abbott, Tokyo, Japan). Those whose' screening test was positive had their status confirmed with a 3^rd ^generation immunochromatographic test (HEXAGON HIV, Human Laboratory, Wiesbaden, Germany). Lymphocytes count for all participants used flux cytometry methods implemented with BD FASCOUNT automate (BD Biosciences, Le pont de Claix, France). Lipid profile was assessed through enzymatic methods (Linear chemicals, Montgat, Spain) for all patients and included total cholesterol (TC), HDL-cholesterol (HDL-c), LDL-cholesterol (LDL-c) and triglycerides (TG). To this end, blood sample was collected after an overnight fast (12 hours) and centrifuged at 3000 cycles/minute, and the serum obtained was then used for lipids determination. The TC/HDL-c ratio was also calculated. In accordance with the US National Cholesterol Education Program, Adult Treatment Panel III (NCEP-ATP III) guidelines, abnormal lipid profile was defined as TC ≥ 200 mg/dl, HDL-c < 40 mg/dl, LDL-c ≥ 130 mg/dl, TG ≥ 150 mg/dl and TC/HDL-c ratio ≥ 5 [14].

### Statistical analysis

Sample size was determined assuming a 5% prevalence of total cholesterol > 200 mg/dl in patients ART-naïve patient, a minimum detectable unadjusted odds ratio (OR) of 2, a Type I error of 5% and a power of 80% [11]. Based on the above, the required sample size was 268 participants (134 ART-naïve and 134 ART-treated patients). Data analysis used Statical package for social sciences (SPSS) version 17 for Windows (SPSS, Chicago, IL). Differences in means and proportions for participants' characteristics were assessed using Student t-test and χ^2 ^tests and variants as applicable, and the influence of likely confounders adjusted for through logistic regressions models. Potential predictors consider for inclusion in models were those found to be correlated with lipid abnormalities during ART elsewhere [11,15]. A probability threshold of *P *< 0.05 was set as the threshold of statistical significance.

## Results

### Characteristics of the study population

In all, 138 participants on ART and 138 ART-naïve participants were included. Sixty-eight (49.3%) patients in the ART-naïve group had active tuberculosis and 55 (39.6%) in the ART group had had tuberculosis prior to been started on ART. Meanwhile, no patient in the ART group had active tuberculosis at inclusion in the study. First-line ART regimens were as followed: d4T/3TC/NVP (61 participants), d4T/3TC/EFV (32 participants), AZT/3TC/NVP (31 participants) and AZT/3TC/EFV (14 participants). Therefore among ART patients, 93 (64.4%) participants were on d4T, 45 (32.6%) on AZT, 92 (66.7%) on NVP and 46 (33.3%) on EFV. All regimens included 3TC. ART patients had been on treatment for an average of 30 months (standard deviation: 13). The profile of participants is described in Table 1. There was no significant difference between the ART group and ART-naive group for age (40.6 vs. 38.7 years, p = 0.07), sex distribution (% men: 34.8% vs. 43.5%, p = 0.139) and origin (% urban: 90.6% vs. 92.8%, p = 0.51). ART participants were more likely to have high body mass index (p = 0.001) and high CD4 lymphocytes count (p = 0.016).

**Table 1:** Profile of 138 ART-naïve and 138 first-line ART treated individuals in Yaounde, Cameroon

Characteristics	First-line ART-treated (n = 138)	ART-naïve (n = 138)	p
Men, n (%)	48 (34.8)	60 (43.5)	0.139
Residence, urban, n (%)	125 (90.6)	128 (92.8)	0.513
Mean age, years (SD)	40.6 (8.7)	38.7 (8.7)	0.069
Mean BMI, kg/m^2 ^(SD)	24.6 (4.7)	23.0 (3.6)	0.001
Median CD4 lymphocytes count, per mm^3^ (IQR)	333 (206-459.3)	274.5 (159-408.3)	0.016
Mean duration on ART, months (SD) Active tuberculosis Past history of tuberculosis	30.2 (13.1) 0 55(39.6)	NA 68 (49.3) 0	NA NA NA

ART, antiretroviral therapy; BMI, body mass index; SD, standard deviation; NA, not applicable; IQR, interquartile range.

### Lipid profile and first-line ART treatment

Mean TC and mean LDL-c were higher in patients on ART than in ART-naïve patients (both p < 0.02). There was no significant difference between the two groups for HDL-c and triglycerides (Table 2). The prevalence of lipid profiles abnormalities is depicted in Table 3. The prevalence of TC ≥ 200 mg/dl was 37.7% among ART group and 24.6% in the ART-naïve group (p = 0.013). The equivalents for LDL-c ≥ 130 mg/dl were 46.4% and 21%, p < 0.001; and those for TC/HDL-c ratio ≥ 5 were 35.5% among patients on ART and 18.6% among ART-naïve patients (p < 0.001).

**Table 2:** Value of different lipid parameters between first-line ART group and ART-naïve group in Yaounde, Cameroon

Lipid parameter	First-line ART treated (n = 138)	ART-naïve (n = 138)	p
Total cholesterol, mg/dl	196 (91)	172 (73)	0.018
HDL cholesterol, mg/dl	49 (26)	49 (25)	0.944
LDL cholesterol, mg/dl	133 (89)	100 (68)	0.001
Triglycerides, median, mg/dl (IQR)	134 (98-174)	134 (111-170)	0.331
TC/HDL-cholesterol ratio	5.0 (3.0)	4.3 (3.5)	0.091

Data are mean values (± standard deviation) unless otherwise indicated. ART, antiretroviral therapy; TC, total cholesterol; IQR, interquartile range.

**Table 3:** Prevalence of abnormal lipid levels among first-line ART-treated patients and ART-naïve patients in Yaounde, Cameroon

Lipid parameter	First-line ART-treated n = 138(%)	ART-naïve n = 138(%)	p
Total cholesterol ≥ 200 mg/dl	52 (37.7)	33 (24.6)	0.013
HDL cholesterol < 40 mg/dl	55 (39.9)	51 (37.0)	0.620
LDL cholesterol ≥ 130 mg/dl	64 (46.4)	29 (21.0)	< 0.001
Triglycerides ≥ 150 mg/dl	60 (43.5)	53 (38.4)	0.392
TC/HDL cholesterol ratio ≥ 5	49 (35.5)	19 (13.8)	< 0.001

ART, antiretroviral therapy; TC, total cholesterol.

In multivariable analysis adjusted for age, sex, body mass index, CD4 count and co-infection with tuberculosis, being on ART was significantly and positively associated with raised total cholesterol, LDL-cholesterol and TC/HDL cholesterol. The adjusted odd ratios (95% confidence interval, p-value) ART-treated vs. ART-naïve was 1.82 (1.06-1.12, p = 0.02) for TC ≥ 200 mg/dl; 2.99 (1.74-5.15), p < 0.0001) for LDL-cholesterol ≥ 130 mg/dl and 1.73 (1.04-2.89, p = 0.03) for TC/HDL-cholesterol ≥ 5 (Table 4).

**Table 4:** HIV treatment and other determinants of abnormal lipid profile in HIV-infected individuals in Yaounde, Cameroon

Outcome variables	Explanatory variables	Adjusted OR (95% CI)	P
TC ≥ 200 (mg/dl)	Antiretroviral therapy	**1.82 (1.06-3.12)**	**0.02**
	Sex (Women vs. Men)	0.83 (0.48-1.43)	0.49
	Age > 40 years	1.27 (0.74-2.17)	0.39
	BMI > 25 kg/m^2^	1.05 (0.59-1.86)	0.87
	Active tuberculosis	0.49 (0.28-0.86)	0.01
	CD4 count ≤ 200/mm^3^	2.56 (0.34-20.00)	0.36
LDL-c ≥ 130 (md/dl)	Antiretroviral therapy	**2.99 (1.74-5.15)**	**< 0.0001**
	Sex (Women vs. Men)	1.06 (0.61-1.82)	0.84
	Age > 40 years	1.13 (0.66-1.94)	0.65
	BMI > 25 kg/m^2^	**1.87 (1.07-3.27)**	**0.02**
	Active tuberculosis	0.54 (0.31-0.95)	0.03
	CD4 count ≤ 200/mm^3^	1.92 (0.24-15.74)	0.54
TC/HDL-c ratio ≥ 5	Antiretroviral therapy	**1.73 (1.04-2.89)**	**0.03**
	Sex (Women vs. Men)	**1.88 (1.11-3.20)**	**0.02**
	Age > 40 years	1.49 (0.89-2.50)	0.20
	BMI > 25 kg/m^2^	0.68 (0.40-1.17)	0.16
	Active tuberculosis	1.12 (0.67-1.89)	0.68
	CD4 count ≤ 200/mm^3^	1.78 (0.22-14.43)	0.59

BMI, body mass index; OR, odds ratio; CI, confidence interval; LDL-c, LDL cholesterol;

TC, total cholesterol; HDL-c, HDL cholesterol

Univariable and multivariable logistic regressions models were also used to investigate independent determinants of lipid derangements in the subgroup on ART alone. In both univariable and multivariable analyses, body mass index was the main determinant of raised LDL-c in ART-treated patients (Table 5).

**Table 5:** Determinants of abnormal lipid profile in 138 first-line ART treated individuals in Yaounde, Cameroon

Outcome variables	Explanatory variables	OR	p^a^	Ajusted OR	p^b^
		(95%CI)		(95% CI)	
TC ≥ 200 (mg/dl)	Sex (Women vs. Men)	0.99 (0.48-2.04)	0.99	0.96 (0.45-2.03)	0.93
	Age > 40 years	1.38 (0.69-2.76)	0.36	1.41 (0.69-2.91)	0.35
	BMI > 25 kg/m^2^	1.22 (0.59-2.52)	0.59	1.06 (0.49-2.29)	0.88
	Treatment duration > 2 years	2.07 (1.03-4.17)	**0.04**	2.08 (1.01-4.27)	0.05
	d4T vs. AZT regimen	0.75 (0.36-1.56)	0.44	0.78 (0.32-1.81)	0.55
	NVP vs. EFV regimen	0.79 (0.26-2.41)	0.67	0.79 (0.21-2.99)	0.73
	Past history of tuberculosis	0.80 (0.39-1.62)	0.54	0.85 (0.41-1.80)	0.68
LDL-c ≥ 130 (md/dl)	Sex (Women vs. Men)	0.70 (0.33-1,51)	0.32	0.67 (0.32-1.40)	0.29
	Age > 40 years	0.74 (0.36-1.54)	0.38	0.57 (0.28-1.18)	0.23
	BMI > 25 kg/m^2^	2.23 (1.04-4.80)	**0.02**	2.65 (1.24-5.68)	**0.01**
	Treatment duration > 2 years	0.93 (0.45-1.93)	0.83	0.87 (0.42-1.80)	0.71
	d4T vs. AZT regimen	1.73 (0.79-3.78)	0.13	1.71 (0.72-4.06)	0.22
	NVP vs. EFV regimen	1.18 (0.34-4.01)	0.77	0.99 (0.26-3.72)	0.98
	Past history of tuberculosis	1.54 (0.73-3.25)	0.22	1.72 (0.89-3.62)	0.15
TC/HDL-c ratio ≥ 5	Sex (Women vs. Men)	1.79 (0.79-4.14)	0.13	1.61 (0.73-3.54)	0.23
	Age > 40 years	0.67 (0.31-1.43)	0.26	0.72 (0.34-1.50)	0.38
	BMI > 25 kg/m^2^	0.99 (0.45-2.16)	0.97	0.88 (0.41-1.89)	0.74
	Treatment duration > 2 years	1.93 (0.60-2.97)	0.08	1.90 (0.89-4.06)	0.1
	d4T vs. AZT regimen	1.33 (0.60-2.97)	0.44	1.56 (0.66-3.73)	0.31
	NVP vs. EFV regimen	1.01 (0.27-3.58)	1	0.68 (0.17-2.65)	0.58
	Past history of tuberculosis	0.72 (0.34-1.56)	0.37	0.77 (0.37-1.63)	0.5

BMI, body mass index; OR, odds ratio; CI, confidence interval; LDL-c, LDL cholesterol; TC, total cholesterol; HDL-c, HDL cholesterol; p^a^, p value for unadjusted odds ratio; p^b^, p value for adjusted odds ratio

### Lipid abnormalities and ART regimens

**Figure 1:**
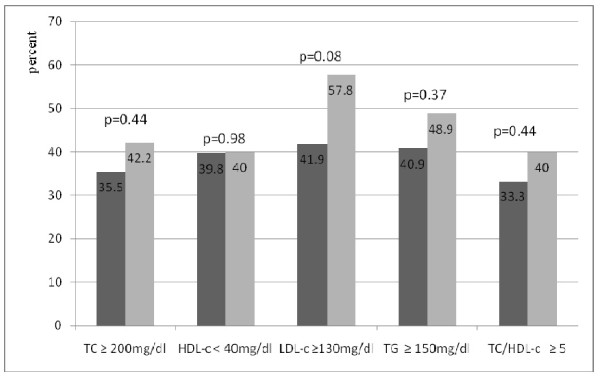
**Prevalence of lipid abnormalities in patients treated with stavudine (left vertical bars) and those treated with zidovudine (right vertical bars)**. ■ Stavudine-based regimen (n = 93). ▩ Zidovudine-based regimen (n = 45). TC, total cholesterol; HDL-c, HDL-cholesterol; LDL-c, LDL-cholesterol; TG, Triglycerides.

**Figure 2:**
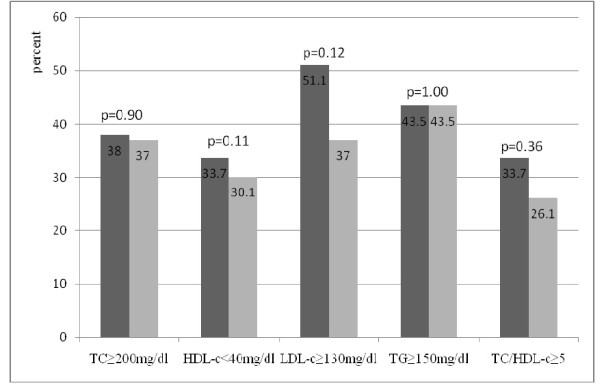
**Prevalence of lipid abnormalities in patients treated with nevirapine (left vertical bars) vs. those treated with efavirenz (right vertical bars)**. ■ Nevirapine-based regimen (n = 92). ▩ Efavirenz-based regimen (n = 46). TC, total cholesterol; HDL-c, HDL-cholesterol; LDL-c, LDL-cholesterol; TG, Triglycerides.

Figures 1 and 2 show the prevalence of lipid abnormalities according to ART regimens. There was no significant difference in the prevalence of lipid abnormalities in patients on regimens that included d4T as compared with those on regimens that included AZT. The prevalence of lipid abnormalities induced by nevirapine and efavirenz was also similar in patients on ART.

## Discussion

The aim of this study conducted in a resource-poor setting was to assess the prevalence of, and characterise lipid abnormalities associated with the use of first-line ART regimens that include nonnucleoside reverse transcriptase inhibitors (NNRTIs). We found that patients on first-line ART had high levels of total cholesterol, LDL-c and high TC/HDL-c ratio as compared with ART-naïve patients. Serum levels of HDL-c and triglycerides were similar between HIV-infected untreated patients and HIV-infected patients on ART. The described derangements that are potentially atherogenic [14], were still present after adjustment for potential confounders.

The association between HAART and adverse lipid profile has been largely described for regimens that include protease inhibitors (PIs) [4,16-19]. It is well known that PIs and NNRTIs induce derangements of lipid profile during ART [3-5,17,20]. However, evidence in support of the adverse effects of NNRTIs on lipid profile of HIV treated patients in sub-Saharan Africa are very limited [11,12]. The proportion of patients with high total cholesterol among our ART treated participant was similar to that reported by Zannou et al in the cohort in Benin [12], but higher than the rate found in rural Ugandans [11]. These two studies were cohort studies wherein participants were followed for a 24 months period for the onset of lipid derangements. However, there are suggestions that the magnitude of first-line ART induced lipid derangements could vary across populations and settings. Cross-sectional studies from other resources-constrained settings have reported prevalence of high level of total cholesterol ranging from 23 to 41% in patients treated with NNRTI-based regimens [15,21]. Based on LDL-c cut-points, we found a prevalence of dyslipidemia that was higher than that reported in Uganda [11] and in India [15]. Prevalence rates in these two studies were respectively 6% and 30%. Similarly, in these two studies the prevalence of high LDL-c prior to ART was also lower than that found in our study (≤ 4% vs. 21%). The HDL-c and triglycerides levels were unaffected by first-line HAART in our study is not in accordance with the findings of Pujari and his colleagues in India [15]. In this study, an 18-month treatment with first-line ART regimens was associated with significant increase in HDL-c and triglycerides.

Several studies have found that stavudine was more involved in the occurrence of lipid derangements as compared with other NRTIs [22,23]. We found no difference in lipid profiles when participants on stavudine were compared to those on zidovudine. This is in line with the findings of Buchacz et al in Uganda [11], and those of Pujari et al in India [15]. The two NNRTIs included in the WHO first-line regimens are nevirapine and efavirenz. Two studies have found that patients treated with nevirapine had a better lipid profile than those treated with efavirenz [24,25]. In accordance with Buchacz et al [11], we found no difference in the lipid profile of patients on nevirapine compared with those on efavirenz. In our study, high LDL-c was associated with ART use, but also with body mass index, including in the subgroup only on ART. The association of high LDL-c with obesity is well known [26]. It is therefore possible that our findings reflect more these well known lipid derangements associated with obesity than sides effects of ART.

Our study did not assess the global cardiovascular risk of participants. However, the increased risk of atherothombotic cardiovascular disease associated with the described lipid derangement is well know [4,5,7,8,27] and would therefore suggest that treatment with first-line ART may actually have harmful effects on the cardiovascular health of our population. Our study, by nature is cross-sectional and inference about causal relationship is not possible. Cohorts' studies would be indicated to monitor lipid profile alterations during first-line ART, and their potential impact on cardiovascular health of people living with HIV in our settings. The other limitation of our study is the lack of HIV-negative controls. In fact untreated HIV infection is associated with low LDL-c and low HDL-c [28], particularly in those patients with severe immune-depression [29]. It is therefore possible that high levels of LDL-c found in our study actually just reflect a "catch-up phenomenon". However, the higher median CD4 count in our HIV-naive subgroup would tend to suggest that they were less likely to have severe immune-depression type lipid profile [29]. In this context, their lipid profile is likely closer to that in the general population without HIV infection. This would also minimise limitations relating to the lack of a non HIV infected control subgroup.

## Conclusion

In conclusion, WHO first-line HAART with regimens that include NNRTIs are associated with potentially atherogenic adverse lipid profile in patients with HIV-1 infection compared to untreated HIV-infected patients in our setting. This may indicate the need to update the current recommendations of WHO pertaining to biological monitoring of patients on first-line antiretroviral therapy to include lipid panel. Lipid profile and other cardiovascular risk factors should be monitored in patients on such therapy so that any untoward effects of ART can be optimally managed.

## List of abbreviations

3TC: lamivudine; ART: antiretroviral therapy; AZT: zidovudine; d4T: stavudine; EFV: efavirenz; HAART: highly active antiretroviral therapy; HDL-c: HDL-cholesterol; HIV: human immunodeficiency virus; LDL-c: LDL-cholesterol; NNRTIs: nonnucleoside reverse transcriptase inhibitors; NVP: névirapine; PIs: protease inhibitors; TC: total cholesterol; TG: triglycerides Statical package for social sciences (SPSS); WHO: World Health Organisation.

## Competing interests

The authors declare that they have no competing interests.

## Authors' contributions

EWPY conceived and designed the study, performed analysis and interpretation of data and drafted the manuscript, AFB and APK assisted with the design, interpretation of data and the critical review of the manuscript. JFKF and JN performed critical review of the manuscript All authors approved and read the final manuscript.
